# Acute Acalculous Cholecystitis as the First Manifestation of Systemic Lupus Erythematosus in a Young Man: A Case Report and Review of Literature

**DOI:** 10.1002/ccr3.71898

**Published:** 2026-02-14

**Authors:** Mohammad Mehdi Shadravan, Arman Ahmadzadeh, Ilad Alavi Darazam, Shahriar Nikpour, Maryam Haghighi Morad, Farid Javandoust Gharebagh, Sadaf Mahmoodkalaye, Setareh Mahmoodi, Legha Lotfollahi

**Affiliations:** ^1^ Student Research Committee Shahid Beheshti University of Medical Sciences Tehran Iran; ^2^ Department of Rheumatology, Loghman Hakim Hospital Shahid Beheshti University of Medical Sciences Tehran Iran; ^3^ Department of Infectious Diseases and Tropical Medicine, Loghman Hakim Hospital Shahid Beheshti University of Medical Sciences Tehran Iran; ^4^ Department of Internal Medicine, Loghman Hakim Medical Center Shahid Beheshti University of Medical Sciences Tehran Iran; ^5^ Department of Radiology, Loghman Hakim Hospital Shahid Beheshti University of Medical Sciences Tehran Iran; ^6^ Infectious Diseases and Tropical Medicine Research Center Shahid Beheshti University of Medical Sciences Tehran Iran; ^7^ Shahid Beheshti University of Medical Sciences Tehran Iran; ^8^ Department of General Surgery, Loghman Hakim Hospital Shahid Beheshti University of Medical Sciences Tehran Iran; ^9^ Department of Nephrology, Loghman Hakim Hospital Shahid Beheshti University of Medical Sciences Tehran Iran

**Keywords:** acalculous cholecystitis, case reports, differential diagnosis, systemic lupus erythematosus

## Abstract

Acute acalculous cholecystitis can rarely be the initial manifestation of systemic lupus erythematosus. Awareness of this atypical presentation is essential to avoid unnecessary surgical intervention. Prompt recognition and initiation of immunosuppressive therapy may resolve symptoms and help establish the correct underlying diagnosis.

## 1 Introduction

Systemic lupus erythematosus (SLE) is a chronic, multisystem autoimmune disease with diverse clinical manifestations and a complex pathogenesis. Kidney, skin, central nervous system involvement, and hematological abnormalities are the most common systemic inflammatory manifestations observed in patients with SLE. Another common clinical manifestation of SLE is gastrointestinal involvement, which can be seen in approximately 40%–60% of patients with SLE [1, 2]. Due to timely treatment, early diagnosis of SLE is associated with lower costs, less accumulation of damage, and an improved quality of life. However, a non‐specific presentation, a single classification criterion, or unusual organ involvement may often occur in the early stages of the disease, leading to frequent delays in diagnosis. These unusual SLE manifestations can affect almost any organ and system and concern physicians in all specialties and settings [3].

Acute acalculous cholecystitis (AAC) is a rare gastrointestinal manifestation of SLE characterized by severe gallbladder inflammation in the absence of cystic duct obstruction due to gallstones [4]. Based on a few published case reports on the incidence of AAC in SLE patients (discussed in Section 5), it can be assumed that AAC is a rare manifestation in SLE patients, and its occurrence as the first manifestation of this disease is much rarer. In this study, we present the case of a 39‐year‐old man with AAC as the first manifestation of SLE. To the best of our knowledge, this is the 13th case report reporting the initial manifestation of AAC in SLE patients. To the best of our knowledge, this is the first reported case of AAC presenting as the initial manifestation of SLE in a young man. This case report was based on the CAse REport (CARE) guidelines [5].

## 2 Case History/Examination

A 39‐year‐old man was admitted to the emergency department with a 2‐month history of epigastric pain that had gradually become diffuse across the abdomen. On presentation, he was alert, oriented, and hemodynamically stable. His vital signs were as follows: blood pressure 160/80 mmHg, body temperature 37.2°C, heart rate 75 beats per minute, and respiratory rate 12 breaths per minute.

The abdominal pain was not related to position or physical activity and was exacerbated by the intake of fatty foods. There were no associated symptoms such as nausea, vomiting, diarrhea, fever, or anorexia. The patient reported food intolerance, progressive discomfort, and an unintentional weight loss of 12 kg over 2 months. He also noted noticeable hair thinning during this period. He denied any history of smoking, alcohol, or illicit drug use. Both personal and family medical histories were unremarkable.

Physical examination revealed right upper quadrant abdominal tenderness and bilateral groin erythema. Initial laboratory tests demonstrated bi‐cytopenia, including hemoglobin of 8.8 g/dL and a platelet count of 113,000/μL. Hepatobiliary enzymes and liver function tests were within normal ranges, including AST 42 IU/L, ALT 35 IU/L, total bilirubin 1.2 mg/dL, alkaline phosphatase 213 U/L, and albumin 4.2 g/dL. Serum creatinine was elevated at 1.7 mg/dL. Electrolyte abnormalities included hyponatremia (Na: 133 mEq/L) and hypokalemia (K: 3.2 mEq/L) (Table 1). Urinalysis revealed hematuria (3+) and proteinuria (3+), which was further confirmed by a 24‐h urine protein of 2795 mg.

**TABLE 1:** Laboratory tests on admission.

Parameter	Patient value	Reference value^a^	Parameter	Patient value	Reference value	Parameter	Patient value	Reference value
WBC	5.6 × 10^9^/L	4.5–11 × 10^9^/L	Alb	4.2 g/dL	3.4–5.4 g/dL	PT	15 s	10–14 s
PMNs	65%	30%–70%	CPK	439 μg/L	39–308 μg/L	PTT	30.5 s	27–45 s
MN	26.5%	20%–45%	LDH	507 U/L	Up to 480	INR	1.4	2–3
Hb	8.8 g/dL	11.5–14.5 g/dL	Haptoglobin	1.78 g/L	2–3 g/L	Urea	95 mg/dL	18–55 mg/dL
MCV	84 fL	80–100 f%	Na	133 mEq/L	135–145 mEq/L	Cr	1.7 mg/dL	0.6–1.2 mg/dL
PLT	113,000/μL	150–450/μL	K	3.2 mmol/L	3.5–5.1 mmol/L	Uric acid	4.6 mg/dL	1.9–8.6 mg/dL
ESR	42 mm/h	< 20	Mg	2.5 mg/dL	1.8–2.6 mg/dL	24 h urine V	1300 mL/day	500–2000 mL/day
Reticulocyte	0.5%–1.1%	0.5%–1.5%	Ca	9.3 mg/dL	8.7–11 mg/dL	24 h urine Cr	2093 mg/day	1504–2001 mg/day
PBS (schistocyte), immature WBC	Negative	Negative/positive	P	3.4 mg/dL	2.5–4.5 mg/dL	24 h urine Pr	2795 mg/day	20–150 mg/day
Dysmorphic RBC	Negative	Negative/positive	PH	7.43	7.31–7.44	AST	42 U/L	Up to 43
Serum iron	89 μg/dL	40–175 μg/dL	PCO_2_	24 mmHg	40–52 mmHg	ALT	35 IU/L	Up to 40
TIBC	197 μg/dL	250–450 μg/dL	HCO_3_	36 mEq/L	22–27 mEq/L	ALK	213 IU/L	80–306 IU/L
Ferritin	774 ng/mL	9–308 ng/mL	BS	88 mg/dL	70–179 mg/dL	Bilirubin total‐direct	1.2 mg/dL 0.4 mg/dL	0.1–1.2 mg/dL < 0.3 mg/dL
BC	Negative	Negative/positive	Amylase	55 U/L	< 110	HBs Ag	Negative	Negative/positive
UC	Negative	Negative/positive	Lipase	74 U/L	≤ 60	HBs Ab	< 10	Positive > 10
HIV Ab	Negative	Negative/positive	VDRL	Negative	Negative/positive	HCV Ab	Negative	Negative/positive

Abbreviations: μg, microgram; Alb, albumin; ALT, alanine aminotransferase; AST, aspartate aminotransferase; BC, blood culture; BS, blood sugar; Ca, calcium; CPK, creatine phosphokinase; Cr, creatinine; dL, deciliter; ESR, erythrocyte sedimentation rate; HB, hemoglobin; HBsAb, hepatitis B surface antibody; HBsAg, hepatitis B surface antigen; HCO_3_, bicarbonate; HCV Ab, hepatitis C antibody; HIV Ab, human immunodeficiency virus antibody; INR, international normalized ratio; IU, international unit; K, potassium; L, liter; LDH, lactate dehydrogenase; MCV, mean corpuscular volume; mEq, milliequivalent; Mg, magnesium; mg, milligrams; MN, monocyte; Na, sodium; ng, nanogram; P, phosphorus; PCO_2_, partial pressure of carbon dioxide; PH, potential hydrogen; PLT, platelet; PMNs, polymorphonuclear neutrophils; Pr, protein; PT, prothrombin time; PTT, partial thromboplastin time; RBC, red blood cell; TIBC, total iron‐binding capacity; UC, urine culture; V, volume; VDRL, venereal disease research laboratory; WBC, white blood cell.

^a^ Reference values are based on the normal laboratory ranges of our institutional laboratory (Loghman Hakim Hospital Laboratory, Tehran, Iran). For parameters with age or sex specific variations, the reference ranges provided correspond to those for an adult male, consistent with the characteristics of this patient.

## 3 Differential Diagnosis, Investigations, and Treatment

**FIGURE 1:**
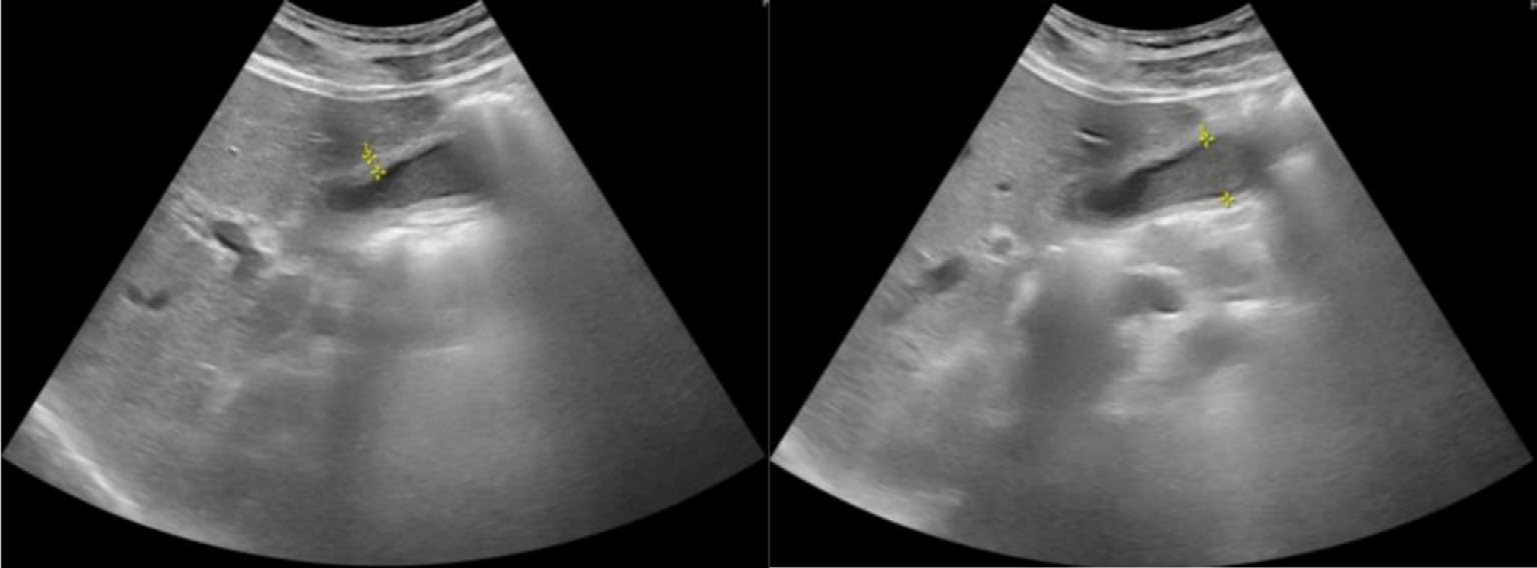
Abdominal ultrasonography demonstrates a gallbladder with significant wall thickening (measuring 15 mm) and the presence of biliary sludge. No gallstones are visualized, and there is no evidence of gallbladder distension.

**FIGURE 2:**
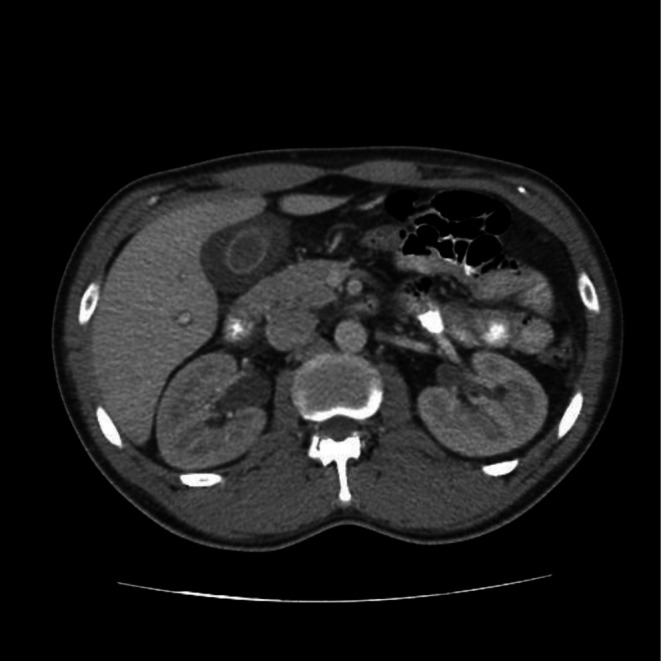
Axial post‐contrast abdominal CT scan showing pericholecystic edema surrounding the gallbladder without evidence of gallstones or significant gallbladder distension.

Abdominal ultrasonography revealed a markedly thickened gallbladder wall (15 mm) and an anterior–posterior diameter of 30 mm, with the presence of biliary sludge but no gallstones or signs of distension (Figure 1). Associated parietal edema and pericholecystic fluid supported the diagnosis of AAC. Contrast‐enhanced computed tomography (CT) confirmed these findings and additionally revealed mild right‐sided ureterohydronephrosis with delayed excretion in the secretory phase (Figure 2). Transthoracic echocardiography demonstrated moderate left ventricular hypertrophy, grade II diastolic dysfunction, moderate‐to‐severe mitral regurgitation with thickened mitral valves, and mild tricuspid regurgitation.

The patient was admitted to the surgical ward with a diagnosis of AAC, and empirical antibiotic therapy was initiated. Cholecystectomy was initially considered; however, further evaluations were prompted due to renal dysfunction, bilateral lower extremity edema, anemia, hematuria, and knee effusion. As a result, consultations with nephrology, hematology, infectious diseases, and rheumatology services were requested. A dermatology consultation was also requested due to mucocutaneous lesions involving the lips, groin, and scrotum. Based on the characteristic appearance of lesions as assessed by the dermatologist, oral acyclovir and topical clotrimazole were empirically prescribed for suspected labial HSV infection and scrotal candidiasis. Throughout the hospitalization, the abdominal pain was intermittent. Laboratory values, including hemoglobin and platelet counts, remained stable during this period.

On hospital day 4, although the patient remained clinically stable and no surgical intervention had been performed, significant proteinuria (2700 mg/24 h) and ongoing systemic findings warranted further evaluation. Consequently, the patient was transferred to the nephrology ward for advanced diagnostic workup and management.

Upon admission to the internal medicine ward, a secondary work‐up for primary and secondary glomerulonephritis was initiated (see Table 2). Given the presence of recurrent, non‐palpable skin rashes, a skin‐punch biopsy was obtained from the lower extremity lesions. Histopathological examination revealed focal parakeratosis, psoriasiform acanthosis, spongiosis, and papillary dermal edema. Additionally, a focal lichenoid interface dermatitis with lymphocytic exocytosis was noted, along with a superficial and deep dermal perivascular inflammatory infiltrate composed of lymphoplasmacytic cells, neutrophils, and eosinophils, accompanied by red blood cell extravasation. These findings were interpreted as compatible with a vasculopathic drug reaction.

**TABLE 2:** Secondary workups for primary and secondary glomerulonephritis.

Parameter	Patient value	Reference value^a^	Parameter	Patient value	Reference value
Rheumatoid factor	Negative	Negative/positive	C4	6.5 mg/dL	10–40 mg/dL
ANA	95	Negative < 20	CH50	108 U/mL	38.7–89.9 U/mL
Anti‐dsDNA Ab	6.9 IU/mL	Negative < 100 IU/mL	Anti‐β2 glycoprotein I (IgG/IgM)	3.7 SGU/1.2 SMU	Negative < 15 U
Anti‐CCP Ab	0.6 EU/mL	Negative < 20 EU/mL	Lupus anticoagulant Ab	Positive (detected)	Normal < 1.20
P‐ANCA (anti‐MPO Ab)	Negative	Negative ≤ 19 AU/mL	Anti‐phospholipid Ab (IgM/IgG)	2.24/0.66 U/mL	Negative < 15 U
C‐ANCA (anti‐PR3 Ab)	Negative	Negative ≤ 19 AU/mL	Anti‐SSA/Ro	198 IU	Negative ≤ 29 AU/mL
C3	67 mg/dL	90–180 mg/dL	Anti‐SSB/La	3 IU	Negative ≤ 29 AU/mL

Abbreviations: ANA, antinuclear antibody; Anti MPO Ab, anti‐myeloperoxidase antibody; Anti‐CCP ab, anti‐cyclic citrullinated peptide antibody; Anti‐dsDNA ab, anti‐double‐stranded deoxyribonucleic acid antibodies; Anti‐PR3 AB, anti‐proteinase‐3 antibodies; Anti‐SSA, anti–Sjögren's‐syndrome‐related antigen A autoantibodies; Anti‐SSB, anti–Sjögren's‐syndrome‐related antigen B autoantibodies; C3, complement component 3; C4, complement component 4; C‐ANCA, antineutrophil cytoplasmic autoantibody; CH50, complement total blood test; dL, deciliter; GLP‐1, glucagon‐like peptide‐1; IgG, immunoglobulin G; IgM, immunoglobulin M; IU, international unit; L, liter; mg, milligram; NL, normal; P‐ANCA, perinuclear anti‐neutrophil cytoplasmic antibodies.

^a^ Reference values are based on the normal laboratory ranges of our institutional laboratory (Loghman Hakim Hospital Laboratory, Tehran, Iran). For parameters with age or sex specific variations, the reference ranges provided correspond to those for an adult male, consistent with the characteristics of this patient.

Although a renal biopsy was initially planned, it was ultimately deferred due to the presence of serologic markers that provided sufficient immunologic evidence to support the diagnosis.

## 4 Conclusion and Results

The diagnosis of SLE in this patient was made based on a combination of clinical manifestations and immunologic findings. Clinically, the patient presented with unexplained AAC, significant proteinuria, anemia, and thrombocytopenia, all suggestive of systemic involvement. Immunologic evaluation revealed positive antinuclear antibodies (ANA), low complement levels, markedly elevated anti‐SSA/Ro antibodies, and a positive lupus anticoagulant (see Tables 1 and 2). Additionally, the presence of antiphospholipid antibodies indicated a possible overlap with secondary antiphospholipid syndrome (APS). The patient was treated with glucocorticoids and cyclophosphamide, and low‐dose aspirin was added due to the elevated lupus anticoagulant.

The patient demonstrated a favorable clinical response to treatment, with resolution of abdominal pain, normalization of serum creatinine levels (1.0 mg/dL), and a follow‐up urinalysis showing no evidence of hematuria or proteinuria.

## 5 Discussion

SLE is a chronic autoimmune disease that affects the entire body. It is observed in women more frequently than in men, with a ratio of almost 10 to 1. SLE is characterized by the presence of ANA, hallmark serological markers of autoimmune dysregulation that contribute to disease pathogenesis through immune complex deposition and inflammation [6]. The disease can trigger a recurrence of symptoms that can be challenging to manage. Since SLE can cause a variety of symptoms, it should be considered a potential cause for many different signs and symptoms. Early identification of SLE is essential for proper treatment and management of the disease. This could help reduce the morbidity and mortality risks associated with the disease [3, 7].

In SLE, gastrointestinal tract involvement is less common than in other systems such as skin, joints, and kidneys. However, it still affects 40%–60% of SLE patients, and the disease can range from mild and non‐specific to severe and life‐threatening. SLE can affect any part of the gastrointestinal tract, from the mouth to the anus. Gastrointestinal problems may arise as a result of SLE, medication side effects, or non‐SLE factors such as infections [8]. A recent multicenter study has also reported that gastrointestinal involvement is common in childhood‐onset SLE, with abdominal pain and elevated liver enzymes being the most frequent clinical findings. These results emphasize the need to consider SLE in the differential diagnosis of gastrointestinal complaints across all age groups [9].

The estimated prevalence of various gastrointestinal diseases in lupus was reported as follows: lupus enteritis (0.59%–10.7%), intestinal pseudo‐obstruction (0.5%–4%), protein‐losing enteropathy (5.5%), and acalculous cholecystitis (0.15%–0.5%) [10]. The causes of these diseases are believed to be small vessel vasculitis, smooth muscle dysfunction, and lymphangiectasia. In this review, it has been reported that AAC is the least common gastrointestinal manifestation in SLE patients. Hence, owing to the limited number of reported cases and inadequate data on diagnosis and treatment, it is imperative to exchange experiences and explore treatment options [10].

Although there have been few reports over the years, most previous reports (Table 3) showed that cholecystitis occurring in SLE patients usually develops during follow‐up of the disease [4, 11, 12, 13, 14, 15, 16, 17]. However, in this study, we presented a case report of a 39‐year‐old man who was hospitalized with AAC. After further investigation, the patient was diagnosed with SLE. Therefore, AAC was identified as the first manifestation of SLE disease in our patient. Based on a retrospective study in China [4], among the 8411 hospitalized SLE patients in Peking Union Medical College Hospital (PUMCH), 13 were identified to have SLE‐AAC. Eleven (84.6%) of participants were female, with a mean age of 30.1 ± 8.6 years. Furthermore, AAC was the first manifestation of SLE in four cases. As a result, the incidence of first‐onset AAC in patients with SLE was less than 0.05%.

**TABLE 3:** Case reports of AAC manifestation in SLE patients.

References	Age (Y‐O)/sex	Country	Duration of SLE	Presentation	Diagnosis of AAC	Treatment/results	Outcome
Chen et al. [11]	46/F	United States	10 years	N/V, abdominal pain Positive Murphy's sign Elevated amylase, lipase, AST, ALT	US, CT: Mass‐like structure in the gallbladder fossa postulated to be a necrotic mass 4.1–6.7 ERCP: Dilated CBD, abscess/inflammatory complex cavity in the region of the gall bladder with a haziness postulated to be a leak	Laparoscopic cholecystectomy	R
Yang et al. (case series) [4]	*N*: 13 (4: AAC as initial presentation) F: 84.6% average age: 30.1 ± 8.6	China	—	Fever, abdominal pain, elevated liver enzymes	CT: Distended gallbladder with a thickened wall, pericholecystic fluid, and no gallstones	GC and other ISs	12 (92.4%) responded to treatment with no relapse, one patient (7.6%) died of septic shock
Kamimura et al. [12]	27/F	Japan	1 month	Epigastric pain and high fever	Positive Murphy's sign US/CT: Pericholecystic edema without gallstones, GB wall thickening	GC	R
Basiratnia et al. [13]	10/M	Iran	3 months	Abdominal pain, N/V, and fever	Positive Murphy's sign US: Gallbladder wall thickening and pericholecystic edema Two non‐shadowing echogenic structures suggesting sludge balls Vasculitis in the medium‐sized arteries of the GB wall with fibrinoid necrosis	GC, CYC, and cholecystectomy	R
Bando et al. [14]	43/F	Japan	3 months	Severe RUQ pain, high‐grade fever	US: GB wall thickening without cholelithiasis Pathological examination: lymphocytic venulitis without arteritis Mesenteric inflammatory veno‐occlusive disease (MIVOD) APS: Negative	GC, laparoscopic cholecystectomy	R
Swanepoel et al. [15]	22/F	South Africa	2 years	Severe cramping abdominal pain, vomiting, tachycardia, pronounced tenderness with guarding and rebound in the epigastrium and RUQ, no fever	Severe edema and adhesions of the GB	GC, CYC and cholecystectomy tube	R
Rozin et al. [16]	61/F	Israel	6 months	Fever, chest pain and progressive dyspnea	US: Thickening of the GB wall, pericholecystic oedema and the absence of gallstones Chest X‐ray; cardiomegaly and left pleural effusion	GC and percutaneous cholecystectomy	R
Shin et al. [17]	39/F	Korea	5 years	Abdominal pain, anorexia, nausea and multiple episodes of diarrhea Tenderness on the RUQ	Positive Murphy's sign CT: Pericholecystic edema, increased thickness of the GB wall without stone	GC	R

Abbreviations: AAC, acute acalculous cholecystitis; ALT, alanine aminotransferase; APS, antiphospholipid syndrome; AST, aspartate aminotransferase; CT, computed tomography; CYC, cyclophosphamide; F, female; GB, gallbladder; GC, glucocorticoid; M, male; *N*, number; N/V, nausea and vomiting; R, remission; RUQ, right upper quadrant; SLE, systemic lupus erythematosus; US, ultra‐sonography; Y‐O, years old.

To the best of our knowledge, in addition to the study by Yang et al. [4], there exist only eight reports regarding the occurrence of AAC as the initial manifestation of SLE in the world [18, 19, 20, 21, 22, 23, 24, 25]. A comprehensive review of these case reports is presented in Table 4. Unlike Table 3, which summarized cases where AAC occurred during the course of established SLE, Table 4 focuses exclusively on those reports describing AAC as the first manifestation of the disease. Yang et al. conducted a study that showed that the initial manifestation of AAC in SLE, although rare, has a significantly higher prevalence in women. According to Table 4, all reported cases were in young women, except for two cases, one in a 12‐year‐old girl and one in a 70‐year‐old woman. All patients, except two who required cholecystectomy, were successfully treated with high‐dose corticosteroids.

**TABLE 4:** AAC as the initial manifestation of SLE.

References	Age (Y‐O)/sex	Chief complaint	Diagnosis of AAC	Fulfilled SLE criteria	Treatment/time to response
Patel et al. [18]	31/F	N/V, anorexia, bloating sensation, generalized weakness, abdominal pain, intermittent fever, discoloration of skin, alopecia, pedal edema/3 months	US: Positive Murphy's sign, mild hepatosplenomegaly, lamellated gall bladder CI‐CT: Increased GB wall thickness, peri GB fluid collectio	6 ACR criteria: Malar rash, discoid rash, oral ulceration, hematological manifestation, anti‐Sm Ab, ANA+	GC and CYC/3–4 days
Lee et al. [19]	24/F	Fever and RUQ pain	US and CT: GB thickening with pericholecystic edema without gallstones or sludge	Discoid rash, hair fragility with broken hairs, pleural effusion, pericardial effusion, proteinuria, leukopenia, thrombocytopenia Immunologic markers: Positive ANA, anti‐dsDNA antibody, antiphospholipid antibody, and low complement levels (10 points from the Systemic Lupus International Collaborating Clinics 2012 criteria and 31 points from the EULAR/ACR 2019 classification criteria)	GC and HCQ/4 days
Eliach et al. [20]	34/F	Two weeks of fever, epigastric pain, nausea, weight loss (10 pounds), dry eyes, dry mouth, and myalgias	CT: Gallbladder wall thickening, without cholelithiasis US confirmed the absence of gallstones	Thrombocytopenia, normocytic anemia, neutropenia. Protein gap and slightly abnormal AST and ALT. Elevated erythrocyte sedimentation rate (ESR) and ferritin (> 4000 ng/mL). Positive ANA, anti‐dsDNA, anti‐Ro, and lupus anticoagulant	GC, plaquenil, AZA
Manuel et al. [21]	20/F	Migratory polyarthralgia, and fever, N/V, RUQ pain, loss of appetite	US: GB slightly distended without wall thickening CT: Increased wall thickness edema around the GB. without stone	Fever, hepatomegaly, splenomegaly, bilateral pleural effusion, pericardial effusion, anemia, leukopenia, hypoalbuminemia, increased ALT, AST, total bilirubin, LDH, ANA+, anti‐dsDNA+, C3, and C4: low, anti‐Sm: 1:80	GC/10 days
Mohapatra et al. [22]	26/F	RUQ pain Two weeks after emergent C/S at 28 weeks	US: GB wall thickening with normal‐sized CBD without stone	Abdominal pain, renal failure, pericardial effusion, immunological testing revealed ANA+, C3 and C4: low Anti‐dsDNA: Neg Renal biopsy: Lupus nephritis (ISN‐RPS class IV‐G)	Cholecystectomy/GB histology: small vessel vasculitis
Choi et al. [23]	70/F	RUQ pain and fever (abrupt onset)	CT: GB wall thickening with pericholecystic edema without stone	5 ACR classification criteria: ANA+, proteinuria, hemolytic anemia, malar rash, pleuritis	GC, CYC and PTGBD/several days after the treatment
Hegarty et al. [24]	30/F	History of fever, diarrhea, and vomiting (5‐day) History of fatigue and flitting polyarthralgia (6‐week) Local tenderness in the RUQ	US: Thick‐walled edematous GB with no dilatation of the common bile duct or intrahepatic ducts	Kidney biopsy: Eosinophilic systemic vasculitis ACR criteria were fulfilled during 2 years with arthritis, rash, anti‐cardiolipin, anti‐dsDNA, and persisting renal abnormalities (proteinuria 1 g/24 h)	GC pulse/AZA
Mendonça et al. [25]	12/F	Fever, abdominal pain, N/V, intense fatigue, loss of appetite, and 10% weight loss over 1 month	Positive Murphy's sign US: Thickening of the GB wall, pericholecystic oedema without gallstones MRI: Confirmed the diagnosis of AAC	Arthritis, photosensitivity, discoid lesions, malar rash, seizures ANA+, anti‐Sm +, possible vasculitis affecting the CNS and the GB, lymph adenomegaly secondary to SLE	GC pulse/AZA

Abbreviations: AAC, acute acalculous cholecystitis; ACR, american college of rheumatology; ALT, alanine aminotransferase; AST, aspartate aminotransferase; AZA, azathioprine; C/S, caesarean; CBD, common bile duct; CI, contrast‐induced; CT, computed tomography; CYC, cyclophosphamide; EULAR, european alliance of associations for rheumatology; F, female; GB, gallbladder; GC, glucocorticoid; HCQ, hydroxychloroquine; IS, immunosuppressive; M, male; MRI, magnetic resonance imaging; N/V, nausea and vomiting; PTGBD, percutaneous transhepatic gallbladder drainage; RUQ, right upper quadrant; SLE, systemic lupus erythematosus; US, ultra‐sonography; Y‐O, years old.

Clinical manifestations, ultrasonography, CT, and hepatobiliary iminodiacetic acid scans were used to establish the diagnosis of AAC [26, 27]. In our patient, the diagnosis was made based on history and physical examination, revealing acute cholecystitis, which was confirmed by ultrasound and contrast‐enhanced CT, despite the absence of stones. As depicted in Figure 2, the mild uretero‐hydronephrosis observed may be related to immune‐mediated inflammation and smooth muscle dysmotility. Although rare, similar mechanisms have been reported in SLE, including small‐vessel vasculitis leading to ureteritis and dysfunction of smooth muscle in the ureter and bladder [28]. Additionally, smooth muscle dysmotility underlying intestinal pseudo‐obstruction has been frequently associated with concomitant hydronephrosis in SLE [29].

SLE is typically classified according to the Systemic Lupus International Collaborating Clinics (SLICC) 2012 and European League Against Rheumatism/American College of Rheumatology (EULAR/ACR) 2019 criteria [30, 31], which were developed for classification rather than direct clinical diagnosis. The diagnosis of SLE in our patient was primarily based on clinical manifestations and serologic findings. Additionally, the patient fulfilled the 2019 EULAR/ACR criteria (positive ANA as the entry criterion and a total weighted score ≥ 10), thereby meeting the definition of SLE under these criteria. This information is included to demonstrate consistency with established classification frameworks and to provide a structured overview of the patient's findings in relation to recognized criteria.

The treatment of SLE in the context of AAC has been the subject of considerable debate. AAC is a disease with high morbidity and mortality and is difficult to diagnose based on clinical and laboratory findings. Historically, cholecystectomy was considered a treatment option due to the significant morbidity associated with AAC [32]. Tables 3 and 4 show that corticosteroid therapy has been effective in SLE cases complicated by AAC. Hydroxychloroquine may reduce the need for corticosteroids and may reduce the risk of organ damage during a flare‐up. The majority of patients achieved remission after treatment without further complications. Nonetheless, there have been instances where individuals who had previously received corticosteroids necessitated a subsequent cholecystectomy [22, 33]. The patient's general health status and risk factors are critical to medical and surgical treatment decisions. Furthermore, our patient responded well to high‐dose corticosteroids, cyclophosphamide, and hydroxychloroquine, so surgery was not required.

SLE is a complex, multifaceted disease characterized by a wide spectrum of clinical manifestations, ranging from mild to severe and potentially life‐threatening. Its heterogeneous nature often complicates timely diagnosis and treatment, frequently leading to suboptimal prognosis and adverse outcomes. Given the disease's unpredictable and diverse presentation, it is essential for clinicians across all medical specialties to remain vigilant for its signs and symptoms. Although atypical manifestations may occur, certain clinical and serologic clues can suggest the underlying diagnosis. Although rare, AAC is a well‐documented manifestation of SLE. Clinicians should consider SLE in patients presenting with this condition, particularly when accompanied by systemic signs or laboratory abnormalities.

## Author Contributions

**Mohammad Mehdi Shadravan:** conceptualization, data curation, writing – original draft, writing – review and editing. **Arman Ahmadzadeh:** investigation, resources, writing – review and editing. **Ilad Alavi Darazam:** investigation, resources, writing – review and editing. **Shahriar Nikpour:** investigation, resources, writing – review and editing. **Maryam Haghighi Morad:** conceptualization, investigation, visualization, writing – original draft. **Farid Javandoust Gharebagh:** investigation, resources, writing – review and editing. **Sadaf Mahmoodkalaye:** methodology, writing – review and editing. **Setareh Mahmoodi:** investigation, resources, writing – review and editing. **Legha Lotfollahi:** conceptualization, project administration, supervision, validation, writing – review and editing.

## Funding

The authors have nothing to report.

## Ethics Statement

This study was approved by the Ethics Committee and Institutional Review Board of Shahid Beheshti University Medical Center, Tehran, Iran (IR.SBMU.RETECH.REC.1402.862, available at: https://ethics.research.ac.ir/EthicsProposalView.php?id=448794).

## Consent

Written informed consent was obtained from the patient for publication of this case report and any accompanying images. A copy of the written consent is available for review by the Editor‐in‐Chief of this journal.

## Conflicts of Interest

The authors declare no conflicts of interest.

## Data Availability

The data that support the findings of this study are available from the corresponding author upon reasonable request. Due to patient privacy and ethical restrictions, the raw clinical data cannot be made publicly available.
